# Study of significance of bone marrow microvessel density in myeloproliferative neoplasms in correlation with CD34 blasts, mast cell count and fibrosis

**DOI:** 10.12688/f1000research.130522.2

**Published:** 2023-10-31

**Authors:** Kesiya Thomas, Ranjitha Rao, Chaithra G V, Sharada Rai, Sneha Rao A R, Kudurugundi Basavaraju Vatsala

**Affiliations:** 1 Department of Pathology, Kasturba Medical College, Mangalore, Manipal Academy of Higher Education, Manipal, Karnataka, India

**Keywords:** myeloproliferative neoplasms, microvessel density, CD34, mast cell tryptase

## Abstract

**Background:** Myeloproliferative neoplasms (MPN) are clonal hematopoietic stem cell diseases characterised by myeloid cell growth from one or more lineages. Angiogenesis, in contrast to other subtypes, plays a substantial role in the pathophysiology of primary myelofibrosis (PMF). Research expressing the correlation of microvessel density (MVD), blasts, fibrosis and mast cell count in MPN cases are rarely conducted. We aimed to study the significance of MVD in correlation with CD34 blasts, mast cells and fibrosis in bone marrow biopsies of MPN patients.

**Methods:** The current research was a cross sectional study conducted on 66 cases diagnosed as MPN during a six-year period. This comprised of 32 chronic myeloid leukemia (CML), 31 PMF and three essential thrombocythemia (ET) cases. Routine staining along with reticulin stain to look for fibrosis and immunohistochemistry (IHC) using CD34 and mast cell tryptase (MCT) were performed.

**Results:** We found increased MVD in PMF, when compared to CML and ET (p = 0.042). Further, mean MVD was observed to be increased with high blast counts (p = 0.036). On follow up, raised mean MVD was seen in those cases with relapse/deceased as compared to disease-free patients, which was highly significant (p = 0.000).

**Conclusions:** Increased MVD score was mostly associated with PMF subtype among all the MPNs. Further, higher MVD was observed to be associated with increased blast count and poor prognosis. With angiogenesis playing a critical role in disease outcome, we now have drugs to regulate angiogenesis that are supported by contemporary research. However, further studies with larger cohorts to establish the theranostic role of MVD in MPNs is recommended.

## Introduction

Myeloproliferative neoplasms (MPN) are a category of disorders characterised by enhanced erythroid, megakaryocytic, or granulocytic cell proliferation. ^1^ The global incidence of MPNs ranges from 1.15 to 4.99 instances per 100,000 individuals. ^2^ In a review article by Titmarsh and others, the highest incidences of chronic myeloid leukaemia (CML) and essential thrombocythemia (ET) were found to be in Europe, but primary myelofibrosis (PMF) was more prevalent in Australia. ^3^ Varma and colleagues found that in India, 231 out of 18,14,298 patients (0.0127%) were diagnosed as MPN, out of which, 207 (89.6%) were CML with BCR-ABL mutation. The remaining MPN cases (n = 24/231, 10.4%) were BCR-ABL negative, the majority of whom were polycythemia vera (PV), which was identified in 11 cases (4.7%), followed by PMF (n = 7, 3%) and ET (n = 6, 2.6%) cases. ^4^

Several studies have demonstrated the significance of neoangiogenesis in tumour growth and progression, but only a few have focused on malignant haematological diseases. ^5^ Angiogenesis is important in the pathophysiology of PMF but less so in PV and ET. In myelofibrosis, transforming growth factor-β (TGFB) and vascular endothelial growth factor (VEGF) are two cytokines that help to enhance the stromal response that entails angiogenesis. The principal focus of angiogenesis is in the bone marrow, which is typically measured as vessel concentration and expressed as MVD. ^6^

Patients with MPN also have higher numbers of bone marrow mast cells. Mast cells originate from multipotent hematopoietic progenitor cells in the bone marrow, but they often do not mature there. Instead, they circulate as immature progenitors via the circulatory system to complete their development peripherally in connective or mucosal tissues. ^7^^,^^8^ Many inflammatory cells surround tumour cells, including mast cells, which can secrete many such proangiogenic factors, leading to endothelial cell proliferation and angiogenesis. ^8^

Studies expressing the significance of MVD in specific MPNs like CML and PMF are done in the past; however, studies to explore the relationship between mast cells, blasts, bone marrow fibrosis and MVD are seldom available in literature. Hence, this study is undertaken to understand the significance of MVD in various MPNs, and to ascertain its relation with blast count, mast cell count and fibrosis along with its role in recovery/prognosis.

## Methods

### Study setting

The current research is a cross sectional study of cases diagnosed over six years duration spanning from January 2016 to December 2022 (five years two months retrospective and 10 months prospective).

### Study design

This study follows ‘The Strengthening the Reporting of Observational Studies in Epidemiology (STROBE) statement guidelines. A completed STROBE checklist can be found in the reporting guidelines. ^9^

### Study sample

We included 66 cases diagnosed as MPN during a six-year period. This comprised of 32 chronic myeloid leukemia (CML), 31 PMF and three essential thrombocythemia (ET) cases.

### Ethical consideration

The Institutional Ethics Committee (Reg. No. ECR/541/Inst/KA/2014/RR20; DHR Reg. No. EC/NEW/INST/2020/742), provided ethical clearance on 17 ^th^ February 2022 with reference no. IEC KMC MLR 12-2020/444 after submitting the proposal letter beforehand. The ethics committee also waived the informed consent as the study is performed on archived tissue blocks.

### Inclusion and exclusion criteria

All bone marrow biopsies diagnosed as MPNs in our laboratory during the study duration were included. Cases in which the blocks and/or slides were not available or the tissue was inadequate for immunohistochemistry (IHC) were excluded.

### Data collection

All previously histomorphologically diagnosed MPNs were retrieved from the Pathology department archives and clinical characteristics, including age, gender, symptoms, signs, laboratory investigations, genetic mutations, treatment and follow up details were gathered from the medical records and patient case record files. A total 66 cases of MPN were studied, out of which 32 cases were CML, 31 cases were PMF and three cases were ET. Bone marrow biopsies were received in 10% neutral buffered formalin fixative. Paraffin embedded tissue blocks were made. Sections of four-micron thickness were prepared using a microtome (Leica RM2255) and stained using haematoxylin and eosin (H&E).

### Haematoxylin and eosin staining procedure

Preparation of Mayer’s hematoxylin was done by dissolving one gram of hematoxylin in one litre of water in a bottle. Aluminium sulphate was then dissolved in a small amount of water in a separate beaker, followed by addition of 0.2 g of sodium iodate to this solution and mixed properly. This was then added to the hematoxylin solution bottle and mixed properly. Finally, 0.2 g of citric acid crystals were added and mixed.

Eosin stain was prepared using Eosin Y powder (10 g) dissolved in distilled water (1000 ml) and 1 ml of glacial acetic acid. The mixture was filtered to get a total of 1000 ml of eosin.

Following section cutting, deparaffinization was caried out first, by incubating the slides in an oven for 40 minutes. They were then cooled and dipped in xylene two times, first for 15 minutes and the second time for 10 minutes. The slides were air dried after which they were dipped in absolute alcohol twice for one minute each. They were then air-dried following which they were dipped in water for five minutes. Following deparaffinisation, sections were washed in tap water. They were dipped for 15 minutes in Mayer’s hematoxylin and kept for 5–10 minutes in running tap water to ensure ‘bluing’. Next, they were dipped for 30 seconds in eosin stain and then washed in tap water, dried, and mounted.

Microscopic examination of the cases was based on morphology according to the World Health Organisation classification as per the data proforma ^9^ by using an Olympus CX 21i microscope. MPNs were typed according to the WHO 2016 classification. This is comprised of chronic myeloid leukemia (CML), BCR-ABL1 positive, chronic neutrophilic leukemia (CNL), polycythemia vera (PV), primary myelofibrosis (PMF) i) Prefibrotic/early stage ii) Overt fibrotic stage, essential thrombocythemia (ET), chronic eosinophilic leukemia, not otherwise specified (NOS), myeloproliferative neoplasm, unclassifiable. ^10^

### Immunohistochemistry (IHC)

Immunohistochemical evaluation with CD34 and mast cell tryptase were done on the archived blocks. IHC staining was performed with CD34 (RTU mouse monoclonal; clone: QBEND/10; PDM050) (BD Biosciences Cat# 550390; RRID:AB_393656) and mast cell tryptase (RTU mouse monoclonal; clone: JC/70A; PDM020) (IMGENEX Cat# IMG-80250; RRID:AB_1152624).

### Immunohistochemistry staining procedure

The tissue taken for IHC was performed on paraffin embedded, formalin fixed samples. A representative block was selected in every case. IHC staining was performed with CD34 (RTU mouse monoclonal; clone: QBEND/10; PDM050) and mast cell tryptase (RTU mouse monoclonal; clone: JC/70A; PDM020) according to the instructions provided by the manufacturer. The secondary antibody used was of Diagnostic Biosystem (Ref: UMR1000PD) against mouse primary antibodies. The staining for IHC was done according to standard procedures.

*Preparation of Tris* (tris hydroxymethyl aminomethane) *EDTA buffer*: Tris buffer, 1.21 g; EDTA disodium salt 0.372 g was dissolved in 1L of distilled water. This was used for antigen retrieval.

*Preparation of stock solution of wash buffer/tris buffer:* Tris buffer and sodium chloride (NaCl) solution were used for preparing the working solution. Tris buffer was prepared by mixing 121.1 g of buffer powder with 1000 ml of distilled water and adjusting the pH to 7.4 by adding hydrochloric acid (HCl) drop by drop. NaCl solution was prepared in a separate beaker by adding 83.33 g of NaCl powder to 1000 ml of distilled water. The working solution was prepared by mixing 30 ml of Tris buffer and 70 ml of NaCl solution in 700 ml of distilled water.

The IHC staining was carried out on 4 μm thick sections of tissue. The tissue sections were incubated on poly L-lysine coated glass slides overnight at 37°C. De-paraffinization was performed by keeping the sections at 60°C for 20 minutes followed by two changes of xylene for 15 minutes each. Slides were dried and alcohol changes for 2 times for 15 minutes each was carried out. The slides were immersed in a solution of 99% methanol and 1% hydrogen peroxide to quench the endogenous peroxidase, for 20 minutes. They were washed in running tap water for 5 minutes.

*Antigen retrieval:* The EDTA buffer was pre-heated for 5 minutes at high power in the microwave oven. After 5 minutes, washed slides were dipped in pre-heated solution for 5 minutes, 10 minutes, and another 5 minutes and allowed to cool. They were washed under running tap water for 5–10 minutes. Slides were dipped in distilled water, then working solution/wash buffer (pH 7.4) for 10 minutes. Next, the slides were incubated for 10 minutes in buffered casein solution with sodium azide to suppress the non-specific binding (power block/peroxidase blocking solution). The slides were incubated with the primary antibody CD34 (RTU mouse monoclonal; clone: QBEND/10; PDM050) and mast cell tryptase (RTU mouse monoclonal; clone: JC/70A; PDM020) for 30 minutes at room temperature. After incubation, they were washed in the working solution for duration of 10 minutes. Next, the slides were treated with Diagnostic Biosystem rabbit/mouse secondary antibody (catalogue number K8002, monoclonal antibody) and incubation was carried out at room temperature. Slides were washed in working solution for 10 minutes and then treated with a freshly prepared solution of 20 μL of 3,3’- Diaminobenzidine (DAB) chromogen and 1000 μl of substrate buffer for 5 minutes to obtain a brown colour. Next, the slides were washed with working solution for 5 minutes. Counterstaining was performed for 2–3 minutes with Meyer’s hematoxylin and slides were rinsed in tap water thereafter. Finally, the slides were dehydrated, cleared and mounted.

*Controls used for IHC:* Positive controls used for respective markers were as follows: CD34, Kidney; Mast cell tryptase, Tonsil

### Assessment of MVD, mast cells, CD34 blasts and reticulin fibrosis

The average number of thin-walled vessels with or without lumen and sinusoids that were detectable by CD34+ endothelial cells were considered to assess MVD. These were then counted in ten hot spot fields and an average was obtained per high power field (HPF). Average MVD in 10 hotspots was scored as follows; 0–7 mvd/10 hotspots – score 1, 8–15 mvd/10 hotspots – score 2, 16–25 mvd/10 hotspots – score 3. ^8^^,^^11^

CD34 is expressed in the cytoplasm of blast cells. For determination of blast percentage on CD34 stained bone marrow biopsy, five representative fields were selected and the number CD34+ cells were counted in a total of five high power fields (40x). ^12^^,^^13^ The scoring was performed as follows; 1–5 blasts/5HPF – score 1, >5–10 blasts/5HPF – score 2, >10–19 blasts/5HPF – score 3. MCT shows cytoplasmic staining in mast cells. Average mast cell numbers were counted using tryptase in 10 oil immersion fields. The grading of mast cells using tryptase was as follows; 3–5 mast cells – 1+, >5–10 mast cells – 2+, >10 mast cells – 3+. ^14^

Reticulin fibrosis was graded according to WHO guidelines, where loose network of intersecting reticulin fibres around the perivascular region was considered grade 1, diffuse and dense reticulin fibres with extensive intersections along with focal bundles of thick collagen fibres as grade 2 and diffuse and dense increase in reticulin fibres with extensive intersections and coarse bundles consistent with thick collagen fibres as grade 3 fibrosis. ^10^

### Statistical analysis

For statistical analysis, SPSS software version 25 (IBM SPSS Statistics (RRID:SCR_016479)) was used. Chi-square test, Fishers exact test, mean MVD and standard deviation were calculated. P-value was used to determine significance of the study. Statistical significance was defined as p-value ≤0.05.

## Results

In a total of 66 cases of MPN studied, 32 (48.48%) were CML, 31 (46.96%) were PMF and three (4.54%) cases were ET. The age range of patients were from 14 to 78 years with the mean age being 51.53 years. The present study found that elderly males were primarily affected and the male to female ratio of 1.9:1.

### Correlation of MVD with bone marrow morphology on biopsy

On morphological assessment of these 66 bone marrow biopsies, the majority of them (n = 59, 89.39%) were hypercellular for age. Increased myelopoiesis was observed in 54 cases (81.81%), out of which left shift was seen in 39 (59%) of them. Erythropoiesis was found to be decreased in 36 (54.54%) cases. Megakaryopoiesis was markedly increased in 62 cases (93.93%), in which 17 (26%) and 8 (12.12%) cases showed MVD scores 2 and 3 respectively. Cases with megakaryocytes in clusters, sheets and diffuse patterns were found to have increased mean MVD (8.85, 8.38 and 11.50 respectively), while the presence of dyspoietic megakaryocytes did not alter the mean MVD significantly. These observations, however, were not statistically significant ( Table 1).

**Table 1.** Correlation of MVD ^1^ with megakaryocytes in MPN ^2^.

Megakaryopoiesis		CD34 MVD			P value (MVD score)	Mean MVD and standard deviation	P value (Mean MVD)
		0–7 MVD/10 hotspots	8–15 MVD/10 hotspots	16–25 MVD/10 hotspots			
Megakaryocytes	Normal	1 (2.6%)	1 (5.6%)	2 (20%)	0.122	11.00 ± 6.68	0.056
	Increased	37 (97.4%)	17 (94.4%)	8 (80%)		7.84 ± 4.28	
Hypolobation	Absent	16 (42.1%)	8 (44.4%)	4 (40%)	0.973	8.50 ± 4.32	0.969
	Present	22 (57.9%)	10 (55.6%)	6 (60%)		7.68 ± 4.57	
Hyperlobation	Absent	31 (81.6%)	13 (72.2%)	8 (80%)	0.722	8.13 ± 4.55	0.495
	Present	7 (81.6%)	5 (27.8%)	2 (20%)		7.64 ± 4.20	
Micro megakaryocytes	Absent	24 (63.2%)	10 (55.6%)	6 (60%)	0.862	7.45 ± 4.42	0.159
	Present	14 (36.8%)	8 (44.4%)	4 (40%)		8.92 ± 4.44	
Dwarf megakaryocytes	Absent	27 (71.1%)	14 (77.8%)	7 (70%)	0.851	8.06 ± 4.47	0.478
	Present	11 (28.9%)	4 (22.2%)	3 (30%)		7.94 ± 4.52	
Clusters	Absent	10 (26.3%)	6 (33.3%)	4 (40%)	0.667	7.67 ± 4.34	0.970
	Present	28 (73.7%)	12 (66.6%)	6 (60%)		8.85 ± 4.72	
Sheets	Absent	22 (57.9%)	14 (77.8%)	4 (40%)	0.128	7.80 ± 4.13	0.411
	Present	16 (42.1%)	4 (22.2%)	6 (60%)		8.38 ± 4.97	
Diffuse	Absent	37 (97.4%)	16 (88.9%)	9 (90%)	0.394	7.81 ± 4.38	0.273
	Present	1 (2.6%)	2 (11.1%)	1 (10%)		11.50 ± 4.80	

^1^ MVD – Microvessel density.

^2^ MPN – Myeloproliferative neoplasms.

### Expression of MVD in different subtypes of MPN

In CML, MVD score 2 and 3 were found only in six (18.75%) and five cases (15.62%) respectively. Out of 31 cases of PMF, 12 (38.7%) cases expressed MVD of score 2, while score 3 was seen in three (9.67%) cases only. In ET, two cases (n = 2/3, 66.66%) expressed MVD of score 3 (p = 0.042). Highest mean MVD was observed in ET (Mean MVD -12.67) compared to CML and PMF (Mean MVD -7.34 and 8.29 respectively) as depicted in Table 2.

**Table 2.** Expression of MVD ^1^ in MPN ^2^ subtypes.

Diagnosis	CD34 MVD			Mean MVD and standard deviation
	0–7 MVD/10 hotspots	8–15 MVD/10 hotspots	16–25 MVD/10 hotspots	
CML ^3^ (n=32)	21 (65.62%)	6 (18.75%)	5 (15.62%)	7.34 ± 4.60
ET ^4^ (n=3)	1 (33.33%)	0 (0.0%)	2 (66.66%)	12.67 ± 5.77
PMF ^5^ (n=31)	16 (51.61%)	12 (38.7%)	3 (9.67%)	8.29 ± 4.02
**P value**	**0.042**			0.127

^1^ MVD – Microvessel density.

^2^ MPN – Myeloproliferative neoplasms.

^3^ CML – Chronic myeloid leukemia.

^4^ ET – Essential thrombocythemia.

^5^ PMF – Primary myelofibrosis.

### Correlation of MVD with bone marrow fibrosis in MPN

**Figure 1.**
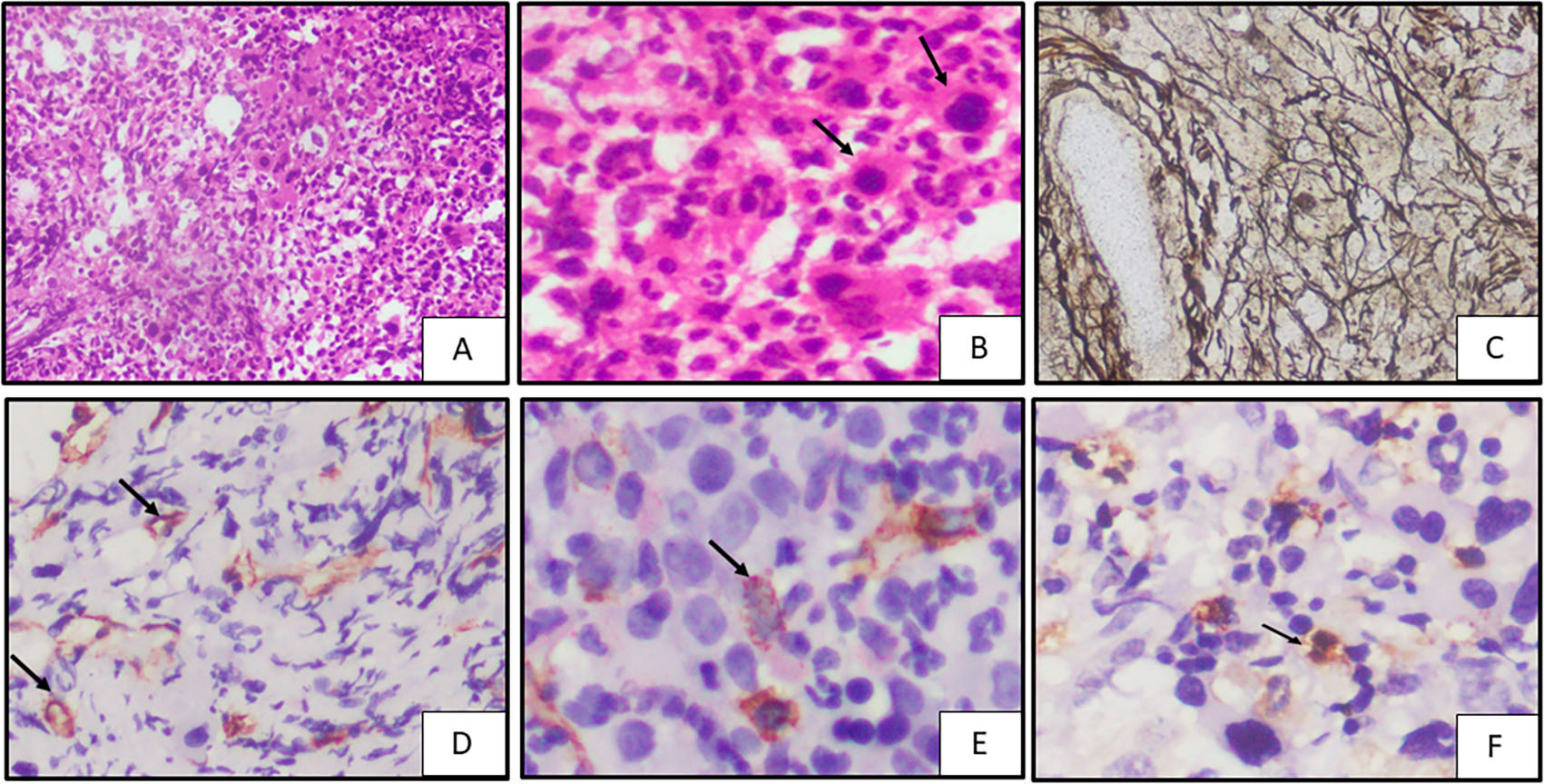
Case of PMF ^1^ on bone marrow biopsy; A. Showing fibrosis (H&E ^2^, 20x); B. Showing hypolobated and micro-megakaryocytes (black arrow) (H&E, 40x); C. Showing WHO ^3^ grade 3 fibrosis (Reticulin stain, 40x); D. CD34 highlights microvessels (black arrow) showing MVD ^4^ score 3 (IHC ^5^, 40x); E. CD34 stains cytoplasm of blasts (black arrow) showing blast score of 1 (IHC, 40x); F. MCT ^6^ highlights cytoplasm of mast cells (black arrow) showing 3+ positivity (IHC, 100x). ^1^PMF – Primary Myelofibrosis, ^2^H&E – Haematoxylin and Eosin stain, ^3^WHO – World Health Organisation, ^4^MVD – Microvessel Density, ^5^IHC – Immunohistochemistry, ^6^MCT – Mast cell tryptase.

Out of 66 cases, 9 (13.63%), 26 (39.39%) and 31 (46.96%) cases were showing grade 1, 2 and 3 fibrosis respectively. In cases with MVD score 2, 66.7% cases were found to have grade 2 fibrosis and in cases with score 3, 50% of them were showing grade 2 fibrosis (p = 0.332) ( Table 3, Figure 1).

**Table 3.** Association of MVD ^1^ with reticulin fibrosis in MPN ^2^.

		CD34 - average MVD in 10 hotspots			Mean MVD and standard deviation
		0–7 mvd/10 hotspots	8–15 mvd/10 hotspots	16–25 mvd/10 hotspots	
Reticulin	Grade 1	5 (13.2%)	2 (11.1%)	2 (20.0%)	8.67±4.95
	Grade 2	17 (44.7%)	4 (22.2%)	5 (50.0%)	7.50±4.88
	Grade 3	16 (42.1%)	12 (66.7%)	3 (30.0%)	8.29±4.02
**P value**		0.332			0.290

^1^ MVD – Microvessel density.

^2^ MPN – Myeloproliferative neoplasms.

### Correlation of MVD with CD34 blasts in MPN

**Figure 2.**
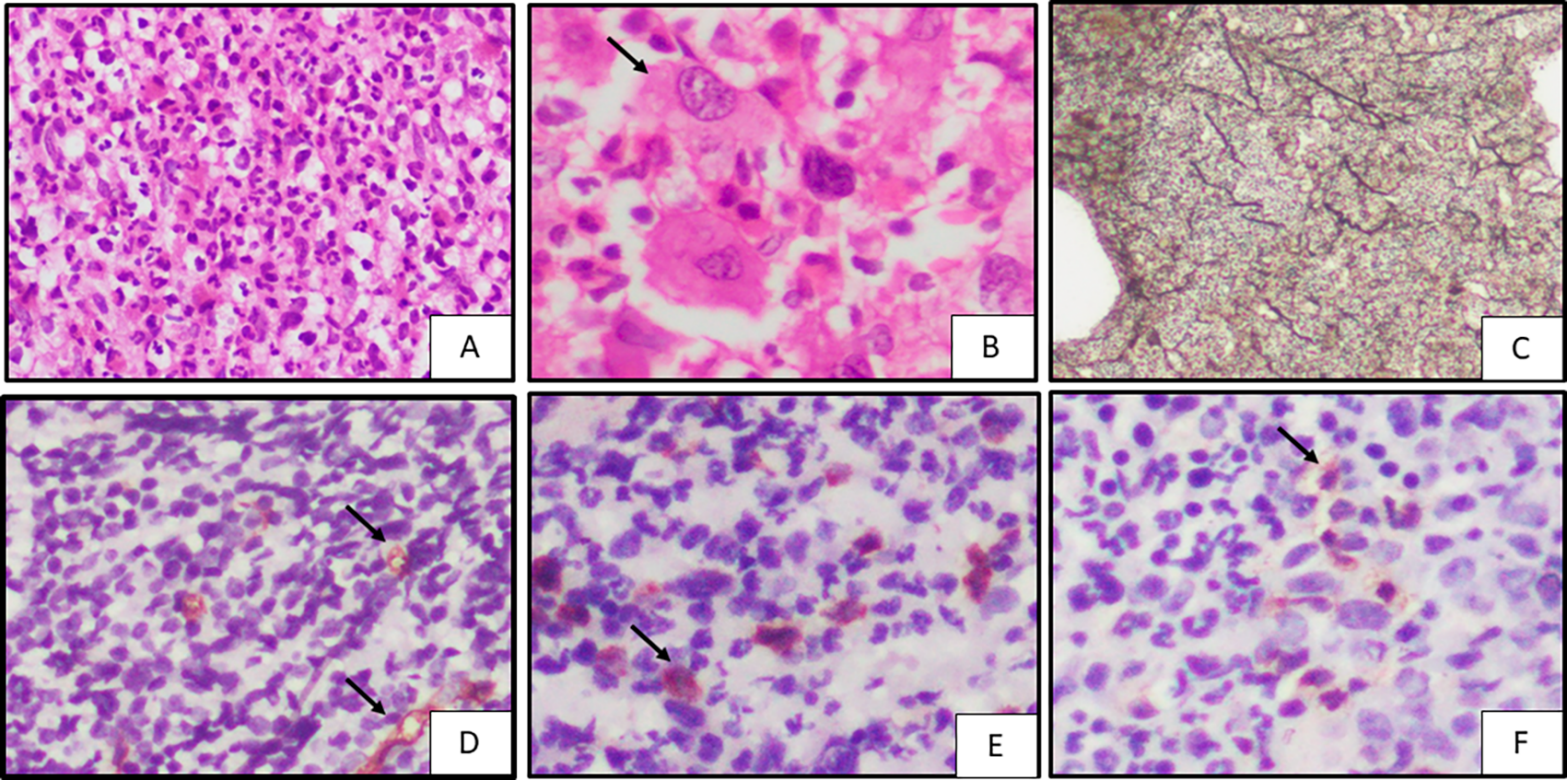
Case of CML ^1^ on bone marrow biopsy; A. Showing left shift (H&E ^2^, 20x); B. Showing ‘dwarf’ megakaryocytes (black arrow) (H&E, 40x); C. Showing WHO ^3^ grade 2 fibrosis (Reticulin stain, 40x); D. CD34 highlights microvessels (black arrow) showing MVD ^4^ score 2 (IHC, 40x); E. CD34 stains cytoplasm of blasts (black arrow) showing blast score of 3 (IHC ^5^, 40x); F. MCT ^6^ highlights cytoplasm of mast cells (black arrow) showing 2+ positivity (IHC, 100x). ^1^CML – Chronic myeloid leukemia, ^2^H&E – Haematoxylin and Eosin stain, ^3^WHO – World Health Organisation, ^4^MVD – Microvessel Density, ^5^IHC – Immunohistochemistry, ^6^MCT – Mast cell tryptase.

When we correlated CD34 blast count score with mean MVD, there was a positive correlation between CD34 blast count and mean MVD ( Table 4). Increased mean MVD was observed with score 2 and 3 of CD34 blasts, which was statistically significant (mean MVD = 8.37 and 10.67 respectively, p = 0.036) ( Figure 2).

**Table 4.** Correlation between MVD ^1^ and CD34 blasts in MPN ^2^.

		CD34 – average MVD in 10 hotspots			Mean MVD and standard deviation
		0–7 mvd/10 hotspots	8–15 mvd/10 hotspots	16–25 mvd/10 hotspots	
CD34-blast In 5HPF ^3^	0 BLASTS	5 (13.2%)	5 (27.8%)	1 (10.0%)	7.73 ± 3.32
	1–5 blasts/5HPF	21 (55.3%)	8 (44.4%)	4 (40.0%)	7.70 ± 4.59
	6–10 blasts/5HPF	11 (28.9%)	4 (22.2%)	4 (40.0%)	8.37 ± 4.81
	11–19 blasts/5HPF	1 (2.6%)	1 (5.6%)	1 (10.0%)	10.67 ± 5.51
**P value**		0.662			**0.036**

^1^ MVD – Microvessel density.

^2^ MPN – Myeloproliferative neoplasms.

^3^ HPF – High power field.

### Correlation of MVD with mast cells in MPN

On analysis, highest mean MVD was seen in cases with mast cell positivity of 3+ and there was a positive correlation between mast cell score and mean MVD. However, we did not get statistical significance with MVD score and mean MVD as seen in Table 5 (p = 0.447).

**Table 5.** Association of MVD ^1^ with Mast cells in MPN ^2^.

Grade (MCT ^3^)		CD34 -average MVD IN 10 hotspots			Mean MVD and standard deviation
		0–7 mvd/10 hotspots	8–15 mvd/10 hotspots	16–25 mvd/10 hotspots	
Average mast cells in 10 oil immersion fields (MCT)	0	3 (7.9%)	0 (0.0%)	0 (0.0%)	3.67 ± 1.53
	1+	25 (65.8%)	9 (50.0%)	5 (50.0%)	7.31 ± 3.92
	2+	9 (23.7%)	8 (44.4%)	4 (40.0%)	9.67 ± 4.67
	3+	1 (2.6%)	1 (5.6%)	1 (10.0%)	10.33 ± 7.51
**P value**	0.447				0.293

^1^ MVD – Microvessel density.

^2^ MPN – Myeloproliferative neoplasms.

^3^ MCT – Mast cell tryptase.

### Correlation of MVD with mutations in MPN

BCR-ABL1 mutation was seen in 32 (48.48%) cases, out of which six (33.3%) and five (50%) cases showed MVD scores 2 and 3 respectively. JAK2 mutation was seen in a total of 32 (48.48%) cases, in which MVD scores of 2 and 3 were found in 11 and five cases respectively. Only five cases showed CALR mutation, out of which only one case showed score 2 MVD as depicted in Table 6. Mean MVD of 8.72 and 6.60 was seen in JAK2 and CALR positive cases respectively (p = 0.053 and 0.059 respectively).

**Table 6.** Association of MVD ^1^ with mutations in MPN ^2^.

Mutations		CD34 – average MVD in 10 hotspots				Mean MVD and standard deviation	P value (mean MVD)
		0–7 mvd/10 hotspots	8–15 mvd/10 hotspots	16–25 mvd/10 hotspots	P value (MVD score)		
BCR-ABL1 ^3^	Absent	17 (44.7%)	12 (66.7%)	5 (50.0%)	0.307	8.59 ± 4.36	0.241
	Present	21 (55.3%)	6 (33.3%)	5 (50.0%)		7.44 ± 4.54	
JAK2 ^4^	Absent	22 (57.9%)	7 (38.9%)	5 (50.0%)	0.411	7.38 ± 4.49	**0.053**
	Present	16 (42.1%)	11 (61.1%)	5 (50.0%)		8.72 ± 4.38	
CALR ^5^	Absent	34 (89.5%)	17 (94.4%)	10 (100.0%)	0.497	8.15 ± 4.59	**0.059**
	Present	4 (10.5%)	1 (5.6%)	0 (0.0%)		6.60 ± 1.95	

^1^ MVD – Microvessel density.

^2^ MPN – Myeloproliferative neoplasms.

^3^ BCR-ABL1 – Breakpoint cluster region – Abelson gene.

^4^ JAK2 – Janus kinase 2.

^5^ CALR – Calreticulin.

### Correlation of MVD with prognosis in MPN

In the present study, all 66 patients were followed up for one year with regular checkups at three-month intervals. Among which, 37 (68.18%) and 17 (15.15%) subjects were rendered disease-free with MVD score of 1 and 2 respectively. Disease relapse was seen in eight cases (12.12%), with the majority (n = 6/8, 75%) expressing MVD score of 3 (p = 0.000) as depicted in Table 7. Thus, mean MVD was higher in those cases with relapse/deceased as compared to disease-free patients.

**Table 7.** Correlation between MVD ^1^ and follow up of patients in MPN ^2^.

		CD34 - average MVD in 10 hotspots			Mean MVD and standard deviation
		0–7 mvd/10 hotspots	8–15 mvd/10 hotspots	16–25 mvd/10 hotspots	
Follow up	Disease free	37 (97.4%)	17 (94.4%)	0 (0.0%)	6.30 ± 2.38
	Relapsed	1 (2.6%)	1 (5.6%)	6 (60.0%)	15.38 ± 3.54
	Deceased	0 (0.0%)	0 (0.0%)	4 (40.0%)	16.75 ± 0.96
**P value**		**0.000**			**0.000**

^1^ MVD – Microvessel density.

^2^ MPN – Myeloproliferative neoplasms.

## Discussion

The present study analysed 66 cases of MPNs, out of which 32 (48.48%) cases of CML, 31 (46.96%) cases of PMF and three (4.54%) cases of ET were considered. Megakaryopoiesis was markedly increased in 62 cases (93.93%), in which 17 (26%) and eight (12.12%) cases showed MVD scores 2 and 3 respectively. Cases with megakaryocytes in sheets and diffuse patterns were found to have increased mean MVD (8.38 and 11.50 respectively), while mean MVD was not majorly altered with respect to dyspoietic megakaryocytes. These observations were not statistically significant. Mesa R. *et al*. observed that increased MVD was associated with clustering of megakaryocytes (p <0.01) in a study conducted in 114 MPN subjects. ^15^ Ponce *et al*. also studied that increased MVD is associated with hypercellularity and megakaryocytic clustering in 56 patients. ^16^

In CML, MVD score 2 and 3 were found only in six (18.75%) and five (15.62%) cases respectively. Out of 31 cases of PMF, 12 (38.7%) cases expressed MVD of score 2, while score 3 was seen in three (9.67%) cases only. In ET, two cases (n = 2/3, 66.66%) expressed MVD of score 3. The highest MVD was observed in ET (mean = 12.67) compared to CML and PMF (mean -7.34 and 8.29 respectively) (p = 0.042). Lundberg L. *et al*. found that MVD was significantly increased by a two-fold degree in CML when compared to normal bone marrow (p <0.01) ^5^ Gianelli U. and colleagues studied 98 cases of Philadelphia negative MPNs and concluded that there were significantly more MVD “hot spots” in PMF (mean ± SD, 25.6 ± 6.3) cases when compared to ET cases (10.1 ± 4.5) and normal control samples (7.5 ± 3.6) (P <0.05). ^17^ These observations were concordant with the findings in our study. Wrobel T. *et al*. found that PMF is the disease with the most evident angiogenesis (increased MVD) and the expression of VEGF positive MVD in bone marrow in PMF patients was significantly higher than in other myeloproliferative disorders. ^18^

Out of 66 cases, nine (13.63%), 26 (39.39%) and 31 (46.96%) cases were showing grade 1, 2 and 3 fibrosis respectively. In cases with MVD score 2, 66.7% cases were found to have grade 2 fibrosis and in cases with score 3, 50% of them were showing grade 2 fibrosis (p = 0.332). Increased MVD at the prefibrotic stage of PMF suggested angiogenesis as an early event signalling the development of fibrosis, according to Boveri *et al*. The study found that the grade of fibrosis increased with increasing MVD. ^6^ Lekovic *et al*. found that CD34-MVD and CD105-MVD strongly (p = 0.001 and p = 0.001, respectively) linked with the grade of bone marrow fibrosis in the entire cohort of MPN patients. ^19^ These observations were in line with the findings of the current study.

When we correlated CD34 blast count score with mean MVD, there was positive correlation between CD34 blast count and mean MVD. Increased mean MVD was observed with score 2 and 3 of CD34 blasts, which was statistically significant (mean MVD = 8.37 and 10.67 respectively, p = 0.036). Padró *et al*. found that increased angiogenesis was seen in 62 patients with acute myeloid leukemia (AML). ^20^ Kuzu *et al*. and Pruneri *et al*. also concluded that increase in marrow micro-vascularity has adverse prognosis in a study carried out on AML patients. ^21^^,^^22^

On analysis, highest mean MVD was seen in cases with mast cell count score of 3 and there was positive correlation between mast cell score and mean MVD. However, we did not get statistical significance (p = 0.447). Xu P. *et al*. found that MVD was increased with increase in mast cells in CML patients, which was in concordance with the present study, but statistically significant. ^8^ Studies on MVD with mast cell association is seldom seen in hematological malignancies.

In the current study, BCR-ABL1 mutation was seen in 32 (48.48%) cases, out of which six (33.3%) and five (50%) cases showed MVD scores 2 and 3 respectively. JAK2 mutation was seen in a total of 32 (48.48%) cases, in which MVD scores of 2 and 3 were found in 11 and five cases respectively. Only five cases showed CALR mutation, out of which only one case showed score 2 MVD. Mean MVD of 8.72 and 6.60 was seen in JAK2 and CALR positive cases respectively (p = 0.053 and 0.059 respectively). Boveri *et al*. and Medinger *et al*, in their study stated that JAK2 mutation was strongly associated with increasing numbers of MVD in all subjects with MPNs. ^6^^,^^13^ Lekovic D. *et al*. also found that MVD was notably raised in patients with JAK2 mutation (CD34-MVD: p = 0.491, p <0.001) but not with CALR mutation. ^19^

In the present study, all 66 patients were followed up for one year with regular check-up at three-month intervals. Among which, 37 (68.18%) and 17 (15.15%) subjects were rendered disease-free with MVD score of 1 and 2 respectively. Disease relapse was seen in eight cases (12.12%), with majority (n = 6/8, 75%) expressing MVD score of 3 (p = 0.000). Thus, mean MVD score was higher in those cases with relapse/deceased as compared to disease-free patients (p = 0.000). Koopmans S. *et al*. in a study conducted on 106 MPN patients found that increased MVD and development of marrow fibrosis correlated with worse prognosis. ^23^ Ponzoni M. *et al*. studied MVD using CD105 and CD34 in 55 MPN patients and observed that MVD was associated with poor prognosis, especially in PMF. ^24^ Pizzi M. *et al*. and Lundberg L. *et al*. also stated the above in their study. ^5^^,^^25^ The above-mentioned study results were concordant with our study.

The present study evaluates bone marrow angiogenesis using MVD score and mean MVD. The p-value obtained using both data have been analysed to draw conclusions between various parameters and we observed that raised MVD score was significantly associated with PMF, while raised mean MVD was found in association with increased CD34 blast counts. Both, MVD score and mean MVD was significant statistically when correlated with disease outcome. Angiogenesis is crucial in the growth and progression of hematopoietic malignancies. ^6^^,^^13^^,^^18^ Anti-angiogenic therapy is primarily an anti-vascular endothelial growth factor (VEGF) or anti-VEGF-receptor (VEGFR) therapy. Several anti-angiogenic drugs, either by themselves or in combination with other anti-tumor therapies, have been licensed for the treatment of these disorders. ^26^

## Conclusions

In the present study, raised MVD is mostly associated with PMF subtype than in other MPNs. In MPNs, cases with increased blast count, mast cell count and fibrosis are also associated with increased MVD. We also found that increased angiogenesis was observed in cases with relapse/deceased, implying the prognostic significance of studying MVD in MPN. With angiogenesis playing a critical role in disease outcome, we now have drugs (bevacizumab) to regulate angiogenesis that are supported by contemporary research.

### Limitations

Out of 66 MPN cases, only three cases of ET and no case of polycythemia vera were evaluated. Hence, further studies with larger cohorts comprising a greater number of each subtype of MPN would be recommended to establish the prognostic/theranostic role of MVD in MPNs.

## Data Availability

Open Science Framework: Underlying data for ‘Study of significance of bone marrow microvessel density in myeloproliferative neoplasms in correlation with CD34 blasts, mast cell count and fibrosis. https://doi.org/10.17605/OSF.IO/E739P. ^9^ This project contains the following underlying data:
• Data.xlsx (Anonymized responses in Excel sheet) • Unedited micrographs: Figures 1a-f and 2a-f.jpg Open Science Framework: Extended data for ‘Study of significance of bone marrow microvessel density in myeloproliferative neoplasms in correlation with CD34 blasts, mast cell count and fibrosis’. https://doi.org/10.17605/OSF.IO/E739P. ^9^ This project contains the following extended data:
• Coding.xlsx (Codes for responses) • Proforma.pdf Open Science Framework: STROBE checklist for ‘Study of significance of bone marrow microvessel density in myeloproliferative neoplasms in correlation with CD34 blasts, mast cell count and fibrosis.’ https://doi.org/10.17605/OSF.IO/E739P. ^9^ Data are available under the terms of the Creative Commons Attribution 4.0 International (CC-BY 4.0)
