# Supplementary material for: Study of significance of bone marrow microvessel density in myeloproliferative neoplasms in correlation with CD34 blasts, mast cell count and fibrosis
Source: F1000Res. 2023 Oct 31;12:503. Originally published 2023 May 16. [Version 2] doi: 10.12688/f1000research.130522.2 (PMC10905004; doi:10.12688/f1000research.130522.2)
Supplement: Peer review history

# Reviewer report: Reviewer response for version 2

**DOI:** 10.5256/f1000research.157527.r219647

**Published:** 2024-02-08

**Authors:** Danijela Lekovic

**Affiliations:** 1 Medical faculty, University of Belgrade, Belgrade, Serbia

Its ok you can accept from my side.

Is the work clearly and accurately presented and does it cite the current literature?

Yes

If applicable, is the statistical analysis and its interpretation appropriate?

I cannot comment. A qualified statistician is required.

Are all the source data underlying the results available to ensure full reproducibility?

Yes

Is the study design appropriate and is the work technically sound?

Yes

Are the conclusions drawn adequately supported by the results?

Yes

Are sufficient details of methods and analysis provided to allow replication by others?

Yes

Reviewer Expertise:

NA

I confirm that I have read this submission and believe that I have an appropriate level of expertise to confirm that it is of an acceptable scientific standard.

# Reviewer report: Reviewer response for version 2

**DOI:** 10.5256/f1000research.157527.r219646

**Published:** 2023-11-07

**Authors:** Anna Rita Migliaccio

**Affiliations:** 1 Unit of Microscopic and Ultrastructural Anatomy, University Campus Bio-Medico, Rome, Italy

I approve the indexing of the paper. Thanks

Is the work clearly and accurately presented and does it cite the current literature?

Partly

If applicable, is the statistical analysis and its interpretation appropriate?

Partly

Are all the source data underlying the results available to ensure full reproducibility?

Partly

Is the study design appropriate and is the work technically sound?

No

Are the conclusions drawn adequately supported by the results?

Partly

Are sufficient details of methods and analysis provided to allow replication by others?

Partly

Reviewer Expertise:

Experimental Hematology including Myeloproliferative neoplasms

I confirm that I have read this submission and believe that I have an appropriate level of expertise to confirm that it is of an acceptable scientific standard.

# Reviewer report: Reviewer response for version 1

**DOI:** 10.5256/f1000research.143288.r197588

**Published:** 2023-09-04

**Authors:** Danijela Lekovic

**Affiliations:** 1 Medical faculty, University of Belgrade, Belgrade, Serbia

The limitations of the study should be noted, that there are no patients with PV and that there is an uneven distribution of patients in the analyzed groups. I think that statistical analysis cannot be done on three patients with ET. I also did not see comparisons with healthy controls. It is also necessary to have the work checked by a statistician because I am not competent to say that it is possible to correlate how it was done.

 After supplementing the work with suggestions, it can be accepted

Is the work clearly and accurately presented and does it cite the current literature?

Yes

If applicable, is the statistical analysis and its interpretation appropriate?

I cannot comment. A qualified statistician is required.

Are all the source data underlying the results available to ensure full reproducibility?

Yes

Is the study design appropriate and is the work technically sound?

Yes

Are the conclusions drawn adequately supported by the results?

Yes

Are sufficient details of methods and analysis provided to allow replication by others?

Yes

Reviewer Expertise:

Myeloproliferative neoplasms

I confirm that I have read this submission and believe that I have an appropriate level of expertise to confirm that it is of an acceptable scientific standard, however I have significant reservations, as outlined above.

# Response

**Published:** 2023-10-13

**Authors:** Ranjitha Rao

**Affiliations:** Kasturba Medical College Hospital, India

All the statistical tests in this study were conducted by a qualified statistician. The number of essential thrombocythemia cases were very few and no polycythemia vera cases were identified during the study and this limitation has been stated after conclusion in the article.

# Reviewer report: Reviewer response for version 1

**DOI:** 10.5256/f1000research.143288.r197587

**Published:** 2023-08-29

**Authors:** Anna Rita Migliaccio

**Affiliations:** 1 Unit of Microscopic and Ultrastructural Anatomy, University Campus Bio-Medico, Rome, Italy

Neoangiogenesis is one of the features of myelofibrosis, the most severe of the Phyladelphia negative myeloproliferative neoplasms and is the subject of extensive investigation. A pubmed search for “angiogenesis” and “myelofibrosis” provided 43 hits.  Methods to quantify angiogenesis in chronic myeloproliferative disorders were published by Thiele (Histil Histopathol 2004, 1245-1260. Angiogenesis was correlated with the expression of vascular endothelial growth factor (likely produced by megakaryocytes) and of its receptor by Medinger et al Br J Haemat 2009, 146:150-157, Boiocchi et al (J Clin Pathol 2011:64, 226 and others. A robust assessment on neoangiogenesis in chronic myeloproliferative disorders (115 patients) was provided by Boveri et al (Br J Haematol 2008, 140: 162 and it is one of the diagnostic criteria for patients with Ph-negative chronic myeloproliferative diseases (Michielis et al Acta Haematol 2015:133:36). Of these papers only that from Boveri is cited in the manuscript.

 Given this extensive background information, the study presented in the paper currently under review correlates the extent of microvessel density with the frequency of CD34+ blasts, mast cells and fibrosis in a cohort of 66 patients with myeloproliferative neoplasms (32 patients with chronic myeloid leukemia, 31 primary myelofibrosis and three essential thrombocythemia) from diagnosis over a period of six years (almost five years retrospectively and 10 months prospectively). The end points were assessed by immune-histomorphology of bone marrow sections with antibodies against CD34 (blasts and endothelial cells) and mast cell tryptase. Based on the data presented the authors conclude that microvessel density is present in patients with Ph-negative myelofibrosis patients and not in patients with BCR-ABL chronic myeloid leukemia and that it frequency correlated with adverse prognosis in myelofibrosis patients.

 Images of the bone marrow two representative patients are presented in Figure 1 (primary myeloprolibrosis) and Figure 2 (chronic myeloproliferative neoplasm). Correlation studies with the data from more patients per group is presented in a table format.

 The Figures are the main problem of the paper: the claimed presence of hypolobulated micromegakaryocytes in Figure 1B and of dwarf megakaryocytes in Figure 2B is not evident. Also not evident are the presence of CD34+ microvessels in 1D and 2D and of CD34+ blasts in 1E and 2E. The quality of the immunohistochemistry is overall disappointing. In addition, normal bone marrow processed in parallel as baseline control is missing.

 Another concern is that it is not clear whether the number of deceased patients is sufficiently high for powerful assessments of adverse factors.

Is the work clearly and accurately presented and does it cite the current literature?

Partly

If applicable, is the statistical analysis and its interpretation appropriate?

Partly

Are all the source data underlying the results available to ensure full reproducibility?

Partly

Is the study design appropriate and is the work technically sound?

No

Are the conclusions drawn adequately supported by the results?

Partly

Are sufficient details of methods and analysis provided to allow replication by others?

Partly

Reviewer Expertise:

Experimental Hematology including Myeloproliferative neoplasms

I confirm that I have read this submission and believe that I have an appropriate level of expertise to confirm that it is of an acceptable scientific standard, however I have significant reservations, as outlined above.

## References

[1] : Bone marrow angiogenesis: methods of quantification and changes evolving in chronic myeloproliferative disorders. *Histol Histopathol* .2004;19(4) : 10.14670/HH-19.1245 1245-60 10.14670/HH-19.1245 15375769

[2] : Angiogenesis and vascular endothelial growth factor-/receptor expression in myeloproliferative neoplasms: correlation with clinical parameters and JAK2-V617F mutational status. *Br J Haematol* .2009;146(2) : 10.1111/j.1365-2141.2009.07726.x 150-7 10.1111/j.1365-2141.2009.07726.x 19466975

[3] : Increased expression of vascular endothelial growth factor receptor 1 correlates with VEGF and microvessel density in Philadelphia chromosome-negative myeloproliferative neoplasms. *J Clin Pathol* .2011;64(3) : 10.1136/jcp.2010.083386 226-31 10.1136/jcp.2010.083386 21217153

[4] : Bone marrow microvessel density in chronic myeloproliferative disorders: a study of 115 patients with clinicopathological and molecular correlations. *Br J Haematol* .2008;140(2) : 10.1111/j.1365-2141.2007.06885.x 162-8 10.1111/j.1365-2141.2007.06885.x 18028479

[5] : Myeloproliferative and thrombotic burden and treatment outcome of thrombocythemia and polycythemia patients. *World Journal of Critical Care Medicine* .2015;4(3) : 10.5492/wjccm.v4.i3.230 10.5492/wjccm.v4.i3.230 PMC452481926261774

# Response

**Published:** 2023-10-13

**Authors:** Ranjitha Rao

**Affiliations:** Kasturba Medical College Hospital, India

The figures are edited as suggested by the reviewer. The hypolobulated micromegakaryocytes in figure 1B, dwarf megakaryocytes in figure 2B are indicated by black arrow.CD34 staining micro vessels and CD34 staining blasts are indicated by black arrow in figure 1D,2D and 1E,2E respectively.

 The statistical tests have been performed by a qualified statistician. The results try to compare the mean vessel density between  those who are deceased of myeloproliferative disease and those who have survived the disease at the time of study. Yes, the number of deceased cases are low and  studies with large number of  cases are required to validate this finding.
